# Southern Medical College

**Published:** 1879-06-20

**Authors:** 


### EDITORIAL AND MISCELLANEOUS.

### *SOUTHERN MEDICAL COLLEGE.*

We go to press with the present number of our Journal in advance of the meeting appointed by the Board of Trustees for the election of a Faculty to the above Institution, and cannot therefore announce the names of the Professors in this issue.

